# COVID-19 and children with Down syndrome: is there any real reason to worry? Two case reports with severe course

**DOI:** 10.1186/s12887-020-02471-5

**Published:** 2020-12-18

**Authors:** Ahmad Kantar, Angelo Mazza, Ezio Bonanomi, Marta Odoni, Manuela Seminara, Ilaria Dalla Verde, Camillo Lovati, Stefania Bolognini, Lorenzo D’Antiga

**Affiliations:** 1 Pediatric Unit, Istituti Ospedalieri Bergamaschi, Gruppo Ospedaliero San Donato, Bergamo, Italy; 2 Pediatric Department, Hospital Papa Giovanni XXIII, Bergamo, Italy; 3 Pediatric Intensive Care Unit, Hospital Papa Giovanni XXIII, Bergamo, Italy

**Keywords:** Down syndrome, Trisomy 21, Coronavirus disease, Children, Case report

## Abstract

**Background:**

Down syndrome (DS) is characterized by a series of immune dysregulations, of which interferon hyperreactivity is important, as it is responsible for surging antiviral responses and the possible initiation of an amplified cytokine storm. This biological condition is attributed to immune regulators encoded in chromosome 21. Moreover, DS is also characterized by the coexistence of obesity and cardiovascular and respiratory anomalies, which are risk factors for coronavirus disease (COVID-19) caused by severe acute respiratory syndrome coronavirus 2 (SARS-CoV-2).

**Case presentation:**

A total of 55 children were admitted to the pediatric ward in Bergamo, between February and May 2020 for COVID-19. Here, we describe the cases of two children with DS and a confirmed COVID-19 diagnosis who had a severe course. In addition, both cases involved one or more comorbidities, including cardiovascular anomalies, obesity, and/or obstructive sleep apnea.

**Conclusions:**

Our observations indicate that children with DS are at risk for severe COVID-19 disease course.

## Background

Down syndrome (DS) is associated with several immune dysregulations [1]. Consequently, the risks of recurrent and severe infections, autoimmune diseases, and inflammatory conditions are commonly reported [2–5]. Immune disorders in DS account for a vast disease burden ranging from quality-of-life issues to more serious health issues and life-threatening issues. Cardiovascular and pulmonary diseases are the most common cause of disease conditions and mortality in DS [6].

The mechanisms by which trisomy 21 causes immune dysregulations in individuals with DS are under continuous investigation. In DS, chromosome 21 harbor multiple genes involved in orchestrating immune responses, and their overexpression induce an overactive immune system. Among the major immune regulators encoded on chromosome 21 are four interferon (IFN) receptors, which serve as receptor subunits for the cytokines interleukin (IL)-10, IL-22, and IL-26 [7]. Furthermore, immune and non-immune cells in patients with DS are hypersensitive to IFN stimulation [1]. Previous studies have shown that T cell lineages of adults with DS show clear signs of heightened activity even in the absence of any obvious infections, a feature that is thought to be caused by chronic IFN hyperactivity [4].

For that reason, the IFN response, which is crucial for escalating antiviral responses, besides driving and amplifying the cytokine storm, is vigorous in people with DS [1, 8].

Recent studies have confirmed that severe acute respiratory syndrome coronavirus 2 (SARS-CoV-2) infections are driven by an exacerbated immune response to the virus, leading an uncontrolled ramping up of the immune response, to a cascade of events involving a cytokine storm, acute respiratory distress syndrome, thromboembolic processes, and multi-organ failure [9].

It is still not clear how individuals with DS respond to this infection. In a recent review, Espinosa provided scientific evidence for considering that individuals with trisomy 21 are at a relatively high risk of developing more severe symptoms and showing increased rates of hospitalization, intensive care admission, secondary bacterial infections, and mortality due to SARS-CoV-2 infection [1]. In addition to immune dysregulation, it is well known that a majority of children with DS are also affected by various congenital anomalies that may increase the risk of severe symptoms. At the initial infection stage, SARS-CoV-2 entry and multiplication could be facilitated in patients with DS due to TMPRSS2 triplication, leading to increased activation of the viral S protein and tight junction downregulation, because tight junctions hamper viral endocytosis [10].

## Case presentation

In Italy, the outbreak of coronavirus disease (COVID-19) started in the Lombardy region, probably on February 18, 2020. The province of Bergamo registered the highest incidence of COVID-19 in Italy. Here, we report the clinical cases of children with DS who presented, with confirmed COVID-19, at the emergency departments of hospitals in Bergamo Province between February and May 2020. One case is a COVID-19 acute viral pneumonia and second case of immunoinflammatory condition that presented nearly 4 weeks after SARS-CoV-2 infection.

### Case 1

A 14-year-old Caucasian girl with DS was admitted to our ward due to fever, cough, nasal congestion, sore throat, fatigue, and dyspnea. She had no cardiac anomalies, but she was overweight with a body mass index (BMI) of 36 and had obstructive sleep apnea (OSA). Before admission, she had already started on amoxicillin-clavulanate and acetaminophen for fever. On admission, chest radiography revealed bilateral interstitial pneumonia. Nasopharyngeal and oropharyngeal swab samples for SARS-CoV-2 were positive. Due to her worsening respiratory condition, she was transferred to the intensive care unit (ICU), where she was initially intubated; then, she was weaned to continuous positive airway pressure (CPAP) and later supported by an oxygen mask. She underwent treatment with antibiotics (ceftriaxone and azithromycin), antiviral drugs (lopinavir and ritonavir), hydroxychloroquine, and low-molecular-weight heparin. Respiratory symptoms recovered after 14 days, and the course was complicated by a sacral bedsore needing prolonged curettage. A lung computed tomography (CT) performed after recovery revealed diffuse ground-glass opacities and bilateral air trapping. No evidence of thromboembolism was found. Spirometry 30 and 60 days after recovery were normal.

### Case 2

A 34-month-old girl of North African origin with DS and a secundum atrial septal defect was admitted to the pediatric ward at the end of January for bilateral interstitial pneumonia, which was managed with external oxygenation using a nasal cannula and antibiotics (ceftriaxone and azithromycin) and required a total of 15 days of hospital stay. No swabs were performed at that time (since it was before the SARS-CoV-2 outbreak in Bergamo). After two weeks of recovery, she was readmitted for high fever and cutaneous rash, which lasted for 3 days, and antibiotic treatment with cefotaxime was initiated. On the third day, she presented with typical signs of Kawasaki disease, such as bilateral non-purulent conjunctival injection, red and cracked lips, strawberry tongue, palmar erythema, peeling of skin from the soles, lymph node enlargement at the neck, and irritability. Hematological examination demonstrated the following parameters: erythrocyte sedimentation rate 51 mm/h, C-reactive protein 14.5 mg/dL, white blood cells 1850 × 10^9^/L, neutrophils 86%, platelets 286 × 10^9^/L, hemoglobin 9.3 gr/dL, hematocrit 31.5%, sodium 129 mEq/L, albumin 2.95 g/dL, aspartate aminotransferase 65 U/L, triglycerides 171 mg/dl; ferritin 640 n/ml, D-dimer 0.7 µg/ml, prothrombin time 93% with international normalized ratio of 1.04, partial thromboplastin time 26 seconds, Fibrinogen 390 mg/dL, lipase 1027 U/L and troponin-I 40 ng/L. Abdominal ultrasound scanning was normal. Electrocardiogram and echocardiogram evaluations of coronary artery aneurysms were normal. Treatment was started according to guidelines of the Italian Society of Pediatrics [11] with intravenous immunoglobulin, oral steroids, and high doses of aspirin, and a second dose of immunoglobulin was administered because of persistent fever. After this treatment, the fever resolved, and aspirin treatment was continued for a total of 6 weeks. Serology for SARS-CoV-2 (IgM, IgG) was positive. Follow-up visits did not reveal cardiac or other organ abnormalities.

## Discussion and Conclusions

Experience with previous coronavirus outbreaks indicates that these viruses have a reduced propensity to affect children. Among patients infected during the 2003 SARS-CoV-1 outbreak, only 6.9% were children, and there were no fatalities in patients aged < 18 years. Additionally, children were found to experience a milder form of the disease [12]. In the Middle East respiratory syndrome coronavirus (MERS-CoV) outbreak, which started in 2012 and still continues, only 2% of the patients were children [13].

At the beginning of this pandemic, children seemed to be relatively spared; however, later reports from various centers described a potentially COVID-19-related severe multisystem inflammatory syndrome in children (MIS-C) and young adults [14, 15].

A study of 46 pediatric COVID-19 patients admitted to ICUs in Canada and the United States found that comorbidities were prevalent in this pediatric cohort, with 50% of patients having one, 17% of patients having two, and 19% of patients having three or more significant comorbidities [16]. Reported comorbidities included medically complex conditions requiring dependence on technological support, immune suppression, malignancy, obesity, diabetes, seizures, sickle cell disease, lung disease, congenital heart disease, and other congenital malformations.

DS is the most common chromosomal abnormality in people worldwide, with a prevalence of approximately 1/1,000 live births. It is characterized by a variety of dysmorphic features, congenital malformations, and other disease conditions. Moreover, DS may be an independent risk factor for thromboembolic disease during childhood with risk of incident cardiovascular events [17, 18].

During the SARS outbreak, no cases involving children with DS were reported. However, during the emergence of MERS-CoV, Menish et al. [19] described a case in a 14-year-old girl with DS with a repaired ventricular septal defect with residual severe mitral regurgitation, a history of systolic and diastolic left ventricular impairment, and pulmonary hypertension. Furthermore, she had obesity (BMI 42.2) as well as hypothyroidism and OSA. On admission, her chest radiography revealed bilateral infiltrates. She was successfully treated with oxygen, nebulization treatment, intravenous diuretics, imipenem, and oseltamivir. She was discharged from the hospital after 7 days.

A confidence study of pediatric COVID-19 cases in 17 pediatric emergency departments in Italy conducted from March 3–27, reported that 27% of the patients had comorbidities. However, it did not report any case of children with DS [20].

Another retrospective cohort study at Children’s National Hospital, Washington DC, included 177 children and young adults with clinical symptoms and laboratory-confirmed SARS-CoV-2 infection who were treated between March 15 and April 30, 2020 [21]. Of the 177 patients who sought medical attention, 44 were hospitalized. Among them, 35 were non-critically ill and nine were critically ill. The study found that cardiac, hematologic, neurologic, and oncologic disorders were more common in hospitalized children with COVID-19 than in non-hospitalized children with the disease. One of the critically ill patients was a 7-week-old girl. She was admitted due to progressive tachypnea and fever, and chest radiography revealed right-lower-lobe pneumonia. Ventilatory support with a RAM cannula was provided.

Literature on COVID-19 in patients with DS is sporadic thus far. Given their characteristics, this population is considered to be at risk [22]. De Cauwer et al. described the clinical course of four adults with DS during the COVID-19 outbreak. In all four patients, the disease course was severe, warranting hospital care in three patients and resulting in a fatal outcome in one [23]. The first case involved a 60-year-old woman with DS who was treated with oxygen and antibiotics (amoxicillin-clavulanate, initially, and meropenem, subsequently) with a favorable outcome. The second case involved a 48-year-old woman who was treated with oxygen, amoxicillin-clavulanate, azithromycin, and chloroquine and recovered. The third case involved a 55-year-old woman who was treated with oxygen, amoxicillin-clavulanate, chloroquine, and azithromycin; however, she did not respond to therapy and died in the hospital. The fourth case involved a 62-year-old patient with DS who developed respiratory failure and subsequently received supportive care. In a recent preprint, Malle et al. reported an analysis of 4,615 patients hospitalized for COVID-19 in the Mount Sinai Health System, within 55 days beginning on March 1, 2020 [24]. They estimated that the incidence of hospitalization in the DS population was 8.9 times higher (CI: 4.00–20.0) than that in the general population, for individuals 30–64 years old. Moreover, these patients were significantly younger than the rest of the hospitalized population.

Kirshan et al. reported the cases of three patients with DS, congenital heart disease, OSA, and pulmonary hypertension who were hospitalized for pneumonia-associated respiratory failure due to SARS-CoV-2 infection [25]. The first case involved a 3-year-old boy with DS, repaired atrioventricular septal defect (AVSD), pulmonary hypertension, OSA with dependence on CPAP, and hypoxic ischemic encephalopathy with seizures. The second case involved a 25-year-old woman with DS, unrepaired AVSD with Eisenmenger physiology, and OSA with bilateral fluffy infiltrates. The third case involved pneumonia-associated respiratory failure in a 21-year man with obesity, OSA, unrepaired partial AVSD with a small primum atrial shunt, and no ventricular shunt.

Since the initial sentinel report from Bergamo Hospital on Kawasaki disease (KD) and KD-like illness related to SARS-CoV-2 infection [15], clinical features of new COVID-19-associated MIS-C have been increasingly observed in children. The clinical features of these cases are both similar and distinct from other well-described inflammatory syndromes in children, including KD. COVID-19-associated MIS-C seems to develop after the infection rather than during the acute stage of COVID-19 [26]. In children, this condition appears to be a severe but delayed immune response to SARS-CoV-2 infection with uncontrolled inflammation presenting with different features such as KD, Kawasaki shock syndrome, toxic shock syndrome or MIS-C and resulting in host organ damage [27]. The causal relationship and pathogenesis of KD and MIS-C remain unclear. Guidance documents based on currently available evidence coupled with expert opinions have been published recently by the American College of Rheumatology [28].

Of the total of 55 children admitted to pediatric wards in Bergamo (between February and May 2020) for SARS-CoV-2 infection, two children with DS had a severe course. The first case is a COVID-19 acute viral pneumonia that needed intubation and respiratory support and second case of immunoinflammatory condition that presented as a complication of SARS-CoV-2 infection. In addition, both cases involved one or more comorbidities, including cardiovascular anomalies, obesity, and/or OSA. Children with DS have a unique profile of cardiovascular diseases. In addition, diverse anatomic abnormalities of the airways are considered as major risk factors for respiratory infections in those with DS. OSA is very common in DS and can trigger pulmonary complications [29]. In the context of COVID-19, obesity is a recognized risk factor. In a recent study, it was reported that 30.4% of hospitalized children had obesity [30]; and obesity is prevalent in patients with DS [31]. This currently presents a challenge in distinguishing the role of comorbidities in the development of COVID-19 in patients with DS. Previous studies have suggested that children with trisomy 21 could be genetically vulnerable to severe SARS-CoV-2 infection [1].

During the COVID-19 outbreak in Italy, the incidence of pediatric emergency visits declined drastically. This observation is attributed to the closure of schools, intensification of public hygiene measures, and wearing of facemasks. These measures for mitigating the spread of COVID-19 seemed to be efficient in protecting not only adults but also children during the peak period. Nevertheless, a few severe infections still occurred in children. As lockdown measures begin to ease and schools reopen in countries still battling the COVID-19 pandemic, it is strongly recommended that sufficient measures are implemented to protect children with DS, particularly those with comorbidities, considering the possibility of COVID-19 resurgence.

## Data Availability

Data sharing is not applicable to this article as no datasets were generated or analyzed during the current study. Data are available from the corresponding author upon request.

## Abbreviations

DS: Down syndrome.
COVID-19: Coronavirus disease 2019
INF: Interferon
IL: Interleukin
SARS-CoV-2: Severe acute respiratory syndrome coronavirus 2 (SARS-CoV-2)
BMI: Body mass index
OSA: Obstructive sleep apnea
ICU: Intensive care unit
CPAP: Continuous positive airway pressure
CT: Computed tomography
MERS-CoV: Middle East respiratory syndrome coronavirus
MIS-C: Multisystem inflammatory syndrome in children
AVSD: Atrioventricular septal defect
KD: Kawasaki disease
